# Rusty pipe syndrome in a lactating mother

**DOI:** 10.11604/pamj.2022.43.169.34281

**Published:** 2022-12-05

**Authors:** Megha Dipak Rudey, Renu Rathi

**Affiliations:** 1 Department of Kaumarbhritya, Mahatma Gandhi Ayurved College Hospital and Research Center, Salod (Hi), Datta Meghe Institute of Medical Sciences, Deemed to be University, Wardha, India

**Keywords:** Rusty pipe syndrome, lactation, bloody discharge, self-limiting

## Image in medicine

Rusty pipe syndrome is a benign physiological condition that causes bilateral bloody discharge in lactating mothers. There is a bloody discharge from the nipples during lactation. It may lead to creating stress or anxiety in mothers, relatives as well as doctors. This is basically a harmless and self-limiting condition. There are various conditions for bloody discharge from nipples, like cracked nipples, mastitis, trauma, and ductal papilloma. This condition occurs during the first week of the lactation period. In this condition the color of breast milk changes to orange-brownish or rusty colored which seems similar to rusty brown water coming from a rusty iron pipe. Here we present the clinical image of rusty pipe syndrome, the rusty brown color which came from the small amount of blood from capillaries, mixes with colostrum or first breast milk. This condition is arising due to the high rate of vascularization of rapidly developing alveoli, which have delicate capillaries. Sometimes these capillaries get ruptured during the early lactation and ooze rusty breast milk. In this condition, feeding should be encouraged, and the mother should be counseled and aware. If the baby tolerates, feeding should continue. This condition is self-limiting and resolves within a week.

**Figure 1:**
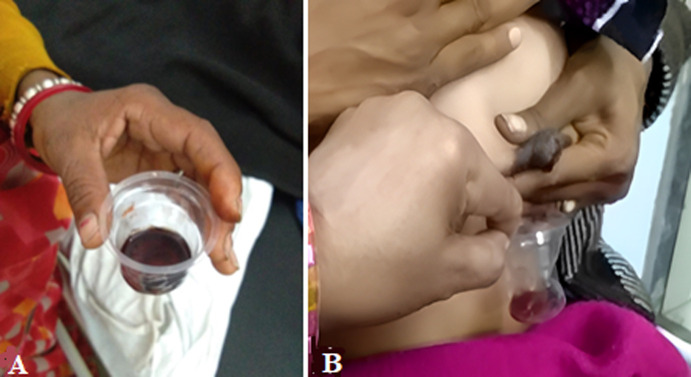
A) plastic glass containing rusty breast milk; B) rusty/bloody discharge from the nipples during lactation

